# Reply to Siddiqui et al., Godbout et al., and Chang and Mao

**DOI:** 10.1093/ajrccm/aamag300

**Published:** 2026-06-24

**Authors:** Geoffrey Chupp, Hiroyuki Nagase, Dirk Skowasch, Gilles Devouassoux, Andréanne Côté, Daniel J Jackson, David J Jackson, Michael E Wechsler, Peter Howarth, Ian D Pavord, all authors of ‘Switching to twice-yearly depemokimab from mepolizumab/benralizumab in severe asthma: a multicenter, randomized, double-blind, phase 3A clinical trial (NIMBLE)’, Geoffrey Chupp, Hiroyuki Nagase, Dirk Skowasch, Gilles Devouassoux, Andréanne Côté, Daniel J Jackson, David J Jackson, Michael E Wechsler, Peter Howarth, Ian D Pavord

**Affiliations:** Department of Internal Medicine, Yale School of Medicine, New Haven, CT, United States; Department of Medicine, Teikyo University School of Medicine, Tokyo, Japan; Department of Internal Medicine II, Cardiology, Pneumology, Angiology, University Hospital Bonn, Bonn, Germany; Service de Pneumologie, Hôpital de la Croix Rousse - HCL, CRISALIS F-CRIN INSERM network, VIRPATH, Université Claude Bernard, Lyon, France; Department of Medicine, Institut Universitaire de Cardiologie et de Pneumologie de Quebec-Université Laval, Quebec City, Canada; Departments of Pediatrics and Medicine, University of Wisconsin-Madison, Madison, WI, United States; Guy’s Severe Asthma Centre, School of Immunology and Microbial Sciences, Guy’s Hospital, King’s College London, London, United Kingdom; Department of Medicine, National Jewish Health, Denver, CO, United States; Global Medical Affairs, GSK, London, United Kingdom; Nuffield Department of Clinical Medicine, University of Oxford, Oxford, United Kingdom

*From the Authors:*

We appreciate the interest in our study, “*Switching to twice-yearly depemokimab from mepolizumab/benralizumab in severe asthma: a multicenter, randomized, double-blind, phase 3A clinical trial (NIMBLE)*,”^1^ and welcome the opportunity to clarify its interpretation.

NIMBLE represents a novel phase 3A study addressing an important and unstudied clinical question: whether patients with severe asthma who are already well controlled on biologic therapy can maintain disease control after switching to twice-yearly depemokimab. The study used a noninferiority design based on the ratio of annualized rate of exacerbations, given no existing precedent in well-controlled patients. While noninferiority was not demonstrated, no conclusions can be drawn regarding relative efficacy between the treatments.

The letters to the editor query our conclusion that most patients with severe asthma may safely and effectively switch to the ultra-long–acting anti–IL-5 depemokimab from the short-acting biologics mepolizumab and benralizumab. Exacerbation rates were low across both treatment arms (0.57 vs 0.49 exacerbations/year with depemokimab vs mepolizumab/benralizumab), with most participants experiencing no exacerbations. The mean absolute difference in exacerbation rate between participants who switched to depemokimab and those who remained on their prior biologic was small (0.08 exacerbations/year). This corresponds to approximately one additional exacerbation every 12.5 years. Additionally, almost all participants did not experience exacerbations requiring hospitalization and/or an emergency department visit (94% with depemokimab; 95% with active comparator). We believe that the absolute difference in annualized exacerbation rates is not clinically meaningful and note that the *American Journal of Respiratory and Critical Care Medicine* editorialists took a similar view regarding the clinical relevance of this difference.^2^

Subgroup analyses showed that the difference in exacerbation rates was primarily driven by participants switching from benralizumab to depemokimab, where there was a relative increase in exacerbations of 38% when switching from benralizumab to depemokimab vs remaining on benralizumab, as highlighted in the letters to the editor. While this relative difference appears considerable, the absolute difference is small at 0.19 exacerbations/year, corresponding to approximately one additional exacerbation every 5.3 years, assuming that patients’ adherence to therapy in a real-world setting reflects that of a randomized clinical trial. Stepping back to consider the overall findings, exacerbation rates across the prestudy biologic subgroups were low, ranging from 0.48 to 0.67 exacerbations/year. In this context, small absolute differences may appear magnified when expressed as relative changes. Our conclusion that this is not clinically meaningful is also supported by the minimal changes from baseline in other measures of asthma control, including the St George’s Respiratory Questionnaire, Asthma Control Questionnaire-5, and FEV_1_, suggesting that symptomatic and lung function benefit is maintained upon switching.

Several factors may contribute to the observed differences. All participants initiated mepolizumab or benralizumab in real-world practice at least 1 year before NIMBLE; therefore, differences may reflect physician-driven patient selection at biologic initiation. Moreover, information on prior biologic use was not available, including history of treatment failure on anti-IL-5/IL-5 receptor biologics, which is an important consideration when switching treatment and may have contributed to the observed differences. Furthermore, treatment stratification was based solely on prestudy biologic treatment and not on parameters predictive of exacerbations, such as baseline exacerbation rate, percent predicted FEV_1_, and blood eosinophil count. As a result, imbalances in these factors may have influenced subgroup outcomes; the mean number of exacerbations in the prior year was 0.34 in participants who were randomly assigned to remain on mepolizumab/benralizumab and 0.41 in participants who switched to depemokimab. Exploratory analyses of baseline patient characteristics did not identify any clear distinguishing features between those who remained stable in NIMBLE and the small number of participants who did not.

The letters proposed mechanistic explanations, including eosinophil depletion and dosing interval considerations. While the relationship between eosinophil suppression and clinical outcomes is of scientific interest, eosinophil levels alone do not explain the observed findings. As is well known, mepolizumab and benralizumab have different mechanisms of action, with mepolizumab maintaining a low blood eosinophil level while benralizumab depletes blood eosinophils altogether.^3^^,^^4^ Therefore, as anticipated, eosinophil levels increased consistently in participants who switched from benralizumab to depemokimab. However, this increase occurred irrespective of whether participants experienced exacerbations or not. Indeed, despite near-complete eosinophil depletion in participants remaining on benralizumab, exacerbation patterns were similar to those observed in participants who switched. We acknowledge that it would be interesting to assess exacerbation phenotypes during biologic treatment in future studies.

The letters to the editor also queried the dosing interval for depemokimab, suggesting that the optimal length may be shorter than 26 weeks. Sustained efficacy with depemokimab has previously been shown across 26-week dosing periods, with no evidence of waning toward the end of the dosing period, in patients with type 2 asthma in a pooled analysis of the SWIFT-1/-2 studies^5^ and over a 2-year period in a pooled analysis of the SWIFT/AGILE studies.^6^

We agree with the letter authors that shared decision making is essential, including when switching between biologics. In doing so, it is recommended that absolute risk differences should be referenced in communication with patients about treatment decisions, as relative risk estimates may lead to misinterpretation.^7-9^ In real-world settings, biologic adherence is suboptimal,^10^ and more than half of patients with severe asthma discontinue biologic therapy within the first year of treatment.^11^^,^^12^ Biologics with longer dosing intervals have been associated with improved adherence, and better adherence is associated with improved clinical outcomes in real-world studies.^12-14^ NIMBLE provides robust evidence to support informed clinician–patient discussions, particularly in the context of individual treatment preferences, adherence, and treatment burden.

In summary, NIMBLE represents a novel phase 3A study in patients already well controlled on biologic therapy, suggesting that they may switch from a short-acting to a long-acting biologic with maintenance of disease control. These findings highlight the need for nuanced interpretation of comparative outcomes in a low event-rate setting and provide a foundation for patient-centered decision making and future research on biologic switching strategies in severe asthma.

## Supplementary Material

aamag300_Supplementary_Data
